# ﻿Multiple evidence reveals two new species and new distributions of *Calocybe* species (Lyophyllaceae) from northeastern China

**DOI:** 10.3897/mycokeys.103.116605

**Published:** 2024-03-13

**Authors:** Ao Ma, Jia-Jun Hu, Yue-Qu Chen, Xin Wang, Yong-Lan Tuo, Lei Yue, Xue-Fei Li, Dan Dai, Yun-Hui Wei, Bo Zhang, Yu Li

**Affiliations:** 1 School of Life Science, Northeast Normal University, Changchun 130024, China; 2 Engineering Research Centre of Edible and Medicinal Fungi, Ministry of Education, Jilin Agricultural University, Changchun 130118, China; 3 Joint Laboratory of International Cooperation in Modern Agricultural Technology, Ministry of Education, Jilin Agricultural University, Changchun 130118, Jilin Province, China; 4 College of Life Science, Zhejiang Normal University, Jinhua 321004, Zhejiang Province, China; 5 Forestry Resources Protection Institute, Jilin Provincial Academy of Forestry Sciences, Changchun 130033, Jilin Province, China; 6 Institute of Agricultural Applied Microbiology, Jiangxi Academy of Agricultural Sciences, Nanchang 330200, Jiangxi Province, China

**Keywords:** Colorful basidiomata, economic values, habitat, new taxa

## Abstract

The *Calocybe* species possess notable economic and medicinal value, demonstrating substantial potential for resource utilization. The taxonomic studies of *Calocybe* are lacking in quality and depth. Based on the specimens collected from northeast China, this study provides a detailed description of two newly discovered species, namely *Calocybebetulicola* and *Calocybecystidiosa*, as well as two commonly found species, *Calocybedecolorata* and *Calocybeionides*. Additionally, a previously unrecorded species, *C.decolorata*, has recently been discovered in Jilin Province, China. The two newly discovered species can be accurately distinguished from other species within the genus *Calocybe* based on their distinct morphological characteristics. The primary distinguishing features of *C.betulicola* include its grayish-purple pileus, grayish-brown to dark purple stipe, smaller basidiomata, absence of cellular pileipellis, and its habitat on leaf litter within birch forests. *Calocybecystidiosa* is distinguished by its growth on the leaf litter of coniferous forests, a flesh-pink pileus, a fibrous stipe with a white tomentose covering at the base, non-cellular pileipellis, larger basidiospores, and the presence of cheilocystidia. The reconstruction of phylogenetic trees using combined ITS, nLSU, and *tef1-α* sequences, employing maximum likelihood and Bayesian inference analyses, showed that *C.betulicola* formed a cluster with *C.decurrens*, while *C.cystidiosa* clustered with *C.vinacea*. However, these two clusters formed separate branches themselves, which also supported the results obtained from our morphological studies. A key to the *Calocybe* species reported from northeast China is provided to facilitate future studies of the genus.

## ﻿Introduction

The genus *Calocybe* Kühner ex Donk is widely distributed in the Northern Hemisphere and has significant economic value. It belongs to the family Lyophyllaceae. However, the genus *Calocybe* is always neglected by researchers. The genus *Calocybe* was officially published in 1962 and is typified by *Calocybegambosa* (Fr.) Donk (Donk 1962). At first, it was treated as a section of *Lyophyllum* P. Karst. (Kühner and Romagnesi 1953). Then, Singer (1962) elevated it to genus rank based on the obvious colorful pileus, separated it from *Lyophyllum*, and belongs to the family Lyophyllaceae. Moreover, Singer divided Calocybe into five sections, namely Sect. Calocybe Singer, Sect. Echinosporae Singer, Sect.Heterosporae Singer, Sect. Pseudoflammulae Singer, and Sect. Carneoviolaceae Sing, by the combination of three characterizes, viz. the color of pileus, spores, and types of pileipellis (Singer 1962). Later, Singer (1986) assigned Sect. Heterosporae to the genus *Lyophyllum*, and, thus, the genus *Calocybe* was divided into four sections (Singer 1986).

By applying molecular methods to research *Calocybe*, it was reconfirmed that *Calocybe* is separate from the genus *Lyophyllum* and belongs to the Lyophyllaceae family (Hofstetter et al. 2002; Moncalvo et al. 2002; Matheny et al. 2006; Garnica et al. 2007). However, the taxonomic systems of *Calocybe* were full of arguments. Hofstetter et al. (2002) and Matheny et al. (2006) revealed that *Calocybe* formed a monophyletic group when the combined ITS, nLSU, or mitSSU fragments were used in phylogenetic analysis. Nevertheless, Bellanger concluded from a multi-gene phylogenetic analysis that *Calocybe* forms a monophyletic clade with *Rugosomyces* Raithelh (Bellanger et al. 2015). Vizzini et al. (2015), Li et al. (2017), and Xu et al. (2021a) also conducted a familiar conclusion with Bellanger. Recent research suggests that the genus *Calocybe* could be divided into five main clades. Based on continuous studies, 62 species of *Calocybe* are listed in the Index Fungorum (www.indexfungorum.org, accessed 20 March 2023).

There have been few studies focusing on the taxonomic and molecular studies of the genus *Calocybe* in China until now. Tai (1979) first reported *Calocybe* species from China, however, under the name *Lyophyllumleucophaeatum* (P. Karst.) P. Karst., later, confirmed to be *Calocybegangraenosa* (Fr.) V. Hofst., Moncalvo, Redhead & Vilgalys. Seven species were recorded from China (Mao 2000; Bau et al. 2003; Fan and Bau 2006). Furthermore, a preliminary taxonomic study on *Calocybe* was performed in recent years (Zhou 2022). And recently, more than ten new species have been described in northeast China (Li et al. 2017; Xu et al. 2021a, 2021b; Qi et al. 2022; Mu and Bau 2023), and proposed their perspective on the taxonomic systematic on *Calocybe*. As a result, a total of 19 species of *Calocybe* have been reported, including *Calocybeaurantiaca* X.D. Yu & Jia J. Li, *Calocybebadiofloccosa* J.Z. Xu & Yu Li, and *Calocybecarnea* (Bull.) Donk, etc.

This study aims to describe and illustrate two new species, one new record from Jilin Province, and one common species based on both morphological and molecular data. Additionally, a key to the reported *Calocybe* species from northeast China is provided.

## ﻿Materials and methods

### ﻿Sampling and morphological studies

The studied specimens were photographed in situ. The size of the basidiomata was measured when fresh. After examination and description of the fresh macroscopic characters, the specimens were dried in an electric drier at 40–45 °C (Hu et al. 2022a, 2022b).

Descriptions of the macroscopic characteristics were based on field notes and photographs, with the colors corresponding to the Flora of British fungi: colour identification chart (Royal Botanic Garden 1969). The dried specimens were rehydrated in 94% ethanol for microscopic examination, and then mounted in 3% potassium hydroxide (KOH), 1% Congo red (0.1 g Congo red dissolved in 10 mL distilled water), and Melzer’s reagent (1.5 g potassium iodide, 0.5 g crystalline iodine, and 22 g chloral hydrate dissolved in 20 mL distilled water) (César et al. 2018); they were then examined with a Zeiss Axio lab. A1 microscope at magnifications up to 1000 ×. All measurements were taken from the sections mounted in the 1% Congo red. For each specimen, a minimum of 40 basidiospores, 20 basidia, 20 cheilocystidia, and 20 widths of pileipellis were measured from two different basidiomata. When reporting the variation in the size of the basidiospores, basidia, cheilocystidia, and width of the pileipellis, 5% of the measurements were excluded from each end of the range, and are given in parentheses. The basidiospores measurements are given as length × width (L × W). Q denotes the variation in the ratio of L to W among the studied specimens, Qm denotes the average Q value of all the basidiospores ± standard deviation. The specimens examined have been deposited in the Herbarium of Mycology of Jilin Agricultural University (HMJAU).

### ﻿DNA extraction, PCR amplification and sequencing

The total DNA was extracted from dried specimens using the NuClean Plant Genomic DNA Kit (Kangwei Century Biotechnology Company Limited, Beijing, China), according to the manufacturer’s instructions. Sequences of the internal transcribed spacer region (ITS), nuclear large ribosomal subunits (nLSU), and translation elongation factor (*tef-1α*) were used for phylogenetic analysis. The ITS sequence was amplified using the primer pair ITS4 and ITS5 (Gardes and Burns 1993), and the nLSU sequence was ampliﬁed using the primer pair LROR and LR5 (Vilgalys and Hester 1990; Cubeta et al. 1991), and *tef1-α* regions were using *tef1-F* and *tef1-R* (Rehner and Samuels 1994). PCR reactions (25 μL) contained dd H_2_O 9.5 μL, 2 × Taq PCR MasterMix 12.5 μL, upstream primer 0.5 μL, downstream primer 0.5 μL, DNA sample 2 μL. Cycle parameters were as follows 2 min at 94 °C; 35 s at 95 °C, 35 s at 48 °C, 1 min at 72 °C for 30 cycles; 10 min at 72 °C; storage at 4 °C (Xu et al. 2021a, 2021b). The PCR products were visualized via UV light after electrophoresis on 1.2% agarose gels stained with ethidium bromide and purified using the Genview High-Efficiency Agarose Gels DNA Purification Kit (Gen-View Scientific Inc., Galveston, TX, USA). The purified PCR products were then sent to Sangon Biotech Limited Company (Shanghai, China) for sequencing using the Sanger method. The new sequences were deposited in GenBank (http://www.ncbi.nlm.nih.gov/genbank; Table 1).

**Table 1.** Voucher/specimen numbers, country, and GenBank accession numbers of the specimens included in this study. Sequences produced in this study are in bold.

Taxa	Gen Bank accession numbers			Voucher/specimen number	Country	References
	ITS	nLSU	*tef1-α*			
*Calocybeaurantiaca*	KU528828	KU528833		SYAU-FUNGI-005	China	Li et al. 2017
*Calocybebadiofloccosa*	NR_173865	MN172334		HMJU:00098	China	Xu et al. 2021a
*Calocybebuxea*	KP885633	KP885625		EB 20140228	Italy	Xu et al. 2021b
***Calocybebetulicola***	**OR771918**	**OR771923**	**OR757443**	**HMJAU48265**	**China**	**This study**
***Calocybebetulicola***	**OR771919**	**OR771924**	**OR757444**	**HMJAU48266**	**China**	**This study**
***Calocybebetulicola***	**OR771920**	**OR771925**	**OR757445**	**HMJAU48267**	**China**	**This study**
*Calocybecarnea*	AF357028	AF223178	DQ367425	CBS552.50	Unknown	Xu et al. 2021a
*Calocybecarnea*	OM905971	OM906008		CC01	Netherlands	Van et al. 2022
*Calocybecarnea*	OQ321901			MQ22-KEG090-HRL3511	Canada	Unpublished
*Calocybecarnea*	MZ159709			K(M):250529	United Kingdom	Unpublished
*Calocybechrysenteron*	KP885640	KP885629		L05-87	Germany	Xu et al. 2021b
*Calocybecoacta*	OK649907	OL687156		HMJU269	China	Xu et al. 2021a
*Calocybeconvexa*	NR_156303	NG_058936		SYAU-FUNGI-008	China	Li et al. 2017
*Calocybecyanella*	MF686498			HMA16	USA	Unpublished
*Calocybecyanea*	OM905975			K(M):56506	Puerto Rico	Unpublished
***Calocybecystidiosa***	**OR771915**		**OR757440**	**HMJAU48268**	**China**	**This study**
***Calocybecystidiosa***	**OR771916**		**OR757441**	**HMJAU48269(1)**	**China**	**This study**
***Calocybecystidiosa***	**OR771917**		**OR757442**	**HMJAU48269(2)**	**China**	**This study**
*Calocybedecolorata*	NR_156302	NG_058938		SYAU-FUNGI-004	China	Li et al. 2017
***Calocybedecolorata***	**OR771922**	**OR771927**		**HMJAU48262**	**China**	**This study**
*Calocybedecurrens*	MT080028	MW444857		HMJU00382	China	Xu et al. 2021a
*Calocybeerminea*	NR_173864	NG_153875		HMJU00100	China	Xu et al. 2019
*Calocybefavrei*	AF357034	AF223183		HAe234.97	Unknown	Xu et al. 2021b
*Calocybefulvipes*	OK649910	OK649880		HMJU03027	China	Xu et al. 2021b
*Calocybegambosa*	AF357027	AF223177		HC78/64	Unknown	Xu et al. 2019
*Calocybegangraenosa*	AF357032	AF223202	DQ367427	Hae251.97	Unknown	Xu et al. 2021a
*Calocybegraveolens*	KP192590			FR2014044	France	Unpublished
*Calocybehebelomoides*	MW672342			HUP-10254	Unknown	Li et al. 2017
*Calocybeindica*	OQ326668	OQ326667		APK2	Unknown	Xu et al. 2021a
*Calocybeionides*	AF357029	AF223179	EF421057	HC77/133	Unknown	Xu et al. 2021a
***Calocybeionides***		**OR771926**	**OR757446**	**HMJAU48264**	**China**	**This study**
*Calocybelilacea*	OM203538	OM341407		SYAU-FUNGI-066	China	Qi et al. 2022
*Calocybelongisterigma*	OM203543	OM341406		SYAU-FUNGI-069	China	Qi et al. 2022
*Calocybenaucoria*	KP192543			FR2013213	France	Xu et al. 2019
*Calocybenaucoria*	KP885642	KP885630		AMB17094	Italy	Xu et al. 2019
*Calocybeobscurissima*	KP192650			BBF-GC01100203	France	Xu et al. 2021a
*Calocybeobscurissima*	KP192652			BBF-GC97111127	France	Bellanger et al. 2015
*Calocybeobscurissima*	MW862295			HBAU15474	China	Unpublished
*Calocybeobscurissima*	OQ133619			HFRG-LG211104-1	United Kingdom	Unpublished
*Calocybeobscurissima*	AF357031	AF223181	EF421058	HC79/181	Unknown	Xu et al. 2021b
*Calocybeochracea*	AF357033	AF223185		BSI94.cp1	Unknown	Bellanger et al. 2015
*Calocybeonychina*	KP192651			FR2014102	France	Bellanger et al. 2015
*Calocybeonychina*	KP192622			FR2014064	France	Bellanger et al. 2015
*Calocybeonychina*	MW084664	MW084704		CAON-RH19-563	USA	Xu et al. 2021b
*Calocybepersicolor*	AF357026	AF223176	EF421059	HC80/99	Unknown	Xu et al. 2019
*Calocybepilosella*	KJ883237			TR gmb 00697	Italy	Floriani and Vizzini 2016
*Calocybepseudoflammula*	MW862362			HBAU15678	Unknown	Unpublished
*Calocybepseudoflammula*	KP192649			FR2014100	France	Bellanger et al. 2015
*Calocybevinacea*	OK649908	OK649876		HMJU5135	China	Xu et al. 2021b
*Lyophyllumatratum*	KJ461896	KJ461895		PDD87010	New Zealand	Xu et al. 2021a
*Lyophyllumcaerulescens*	AF357052	AF223209		HC80.140	Unknown	Xu et al. 2019
*Lyophyllumdecastes*	AF357059	AF042583		JM87/16(T1)	Unknown	Xu et al. 2021b
*Lyophyllumdeliberatum*		MK278318		G0631	Austria	Xu et al. 2019
*Lyophyllumoldea*	OM905959	OM906001	OM974134	BR5020029402116	Unknown	Unpublished
*Lyophyllumsemitale*	AF357049	AF042581		HC85/13	Unknown	Xu et al. 2021b
*Asterophoralycoperdoides*	OM905969	OM906006		AL01	Netherlands	Unpublished
*Asterophoramirabilis*	NR_173484			MEL228691	Unknown	Unpublished
*Asterophoraparasitica*	OM905970	OM906007		AP01	Netherlands	Unpublished
*Hypsizygustessulatus*	KP192623			FR2014065	France	Bellanger et al. 2015
*Hypsizygusulmarius*	EF421105	AF042584		DUKE-JM/HW	Unknown	Unpublished
*Tricholomellaconstricta*	DQ825429	AF223188		HC84/75	Unknown	Xu et al. 2021a
*Tricholomellaconstricta*	JN790692			EC8205	Italy	Unpublished
*Tephrocybeambusta*	AF357058	AF223214		CBS450.87	Unknown	Unpublished
*Tephrocyberancida*	OM905966	OM906004		CORT012400	Unknown	Unpublished
*Tephrocyberancida*	OM905965	OM906003	OM974135	CORT012399	Unknown	Unpublished
*Tephrocyberancida*	OM905967	OM906005	OM974137	TR2017	Unknown	Unpublished
*Tricholomaterreum*	JN389319	JN389374		F130649	Sweden	Unpublished

### ﻿Data analysis

Based on the results of BLAST and morphological similarities, the sequences obtained and related to these samples were collected and are listed in Table 1. The dataset of ITS, nLSU, and *tef1-α* resign comprised sequences from this study, with 67 representative sequences showing the highest similarity to *Calocybe* spp. This dataset included all *Calocybe* species with sequences deposited in GenBank to further explore the relationships of the newly sequenced Chinese specimens within the genus. Moreover, representative species within family Lyophyllaceae were also included to explore the relations within it. The sequences of *Tricholomaterreum* (Schaeff.) P. Kumm. were selected as the outgroup taxon.

Of the dataset, each gene region was aligned using Clustal X (Thonpson et al. 1997), MACSE 2.03 (Ranwez et al. 2018), or MAFFT 7.490 (Katoh and Standley 2013), and then manually adjusted in BioEdit 7.0.5.3 (Hall 1999). The datasets first were aligned, and then the ITS, nLSU, and *tef1-α* sequences were combined with Phylosuite 1.2.2 (Zhang et al. 2020). The best-fit evolutionary model was estimated using Modelfinder (Kalyaanamoorthy et al. 2017). Following the models, Bayesian inference (BI) algorithms were used to perform the phylogenetic analysis. Specifically, BI was calculated with MrBayes 3.2.6 with a general time-reversible DNA substitution model and a gamma distribution for rate variation across the sites (Ronquist and Huelsenbeck 2003). Four Markov chains were run for two runs from random starting trees for two million generations until the split deviation frequency value was < 0.01; the trees were sampled every 100 generations. The first 25% of the sampled trees were discarded as burn-in, while all the remaining trees were used to construct a 50% majority consensus tree and for calculating the Bayesian posterior probabilities (BPPS). RaxmlGUI 2.0.6 (Edler et al. 2021) was used for maximum likelihood (ML) analysis along with 1,000 bootstraps (BS) replicates using the GTRGAMMA algorithm to perform a tree inference and search for the optimal topology. Then the FigTree 1.3.1 was used to visualize the resulting trees.

## ﻿Results

### ﻿Phylogenetic analysis

**Figure 1.**
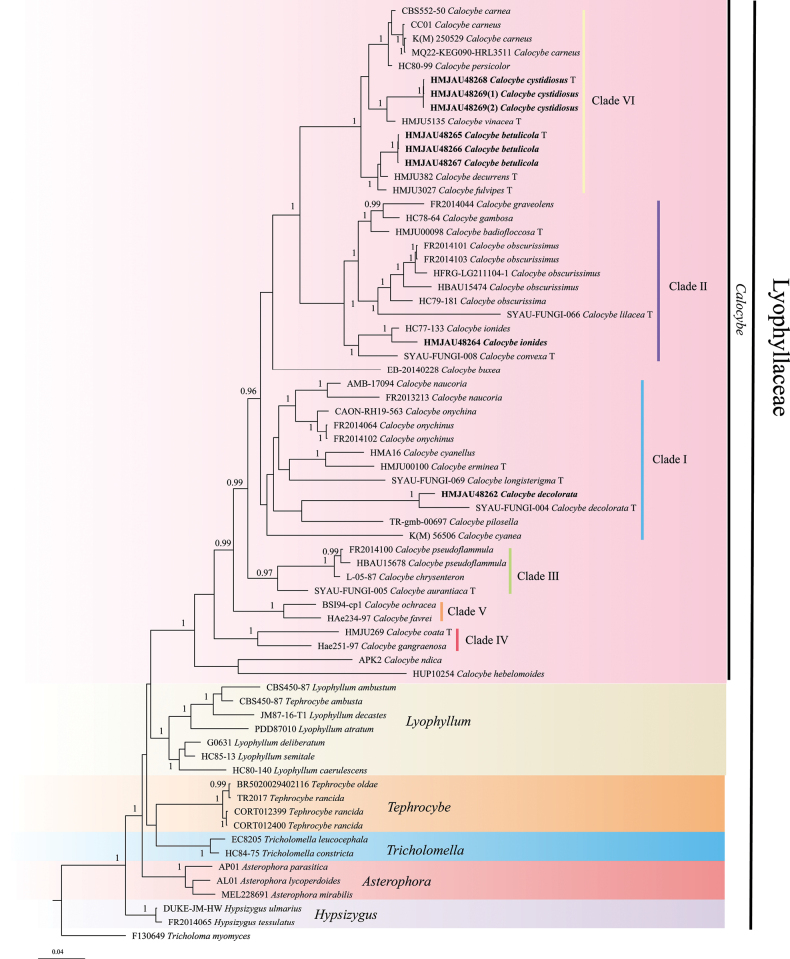
Bayesian analysis phylogenetic tree generated from the ITS, nLSU and *tef1-α* dataset. Bayesian posterior probabilities ≥ 0.95 from BI analysis are shown on the branches. Newly sequenced collections are indicated in bold, and the type specimens are denoted by (T).

**Figure 2.**
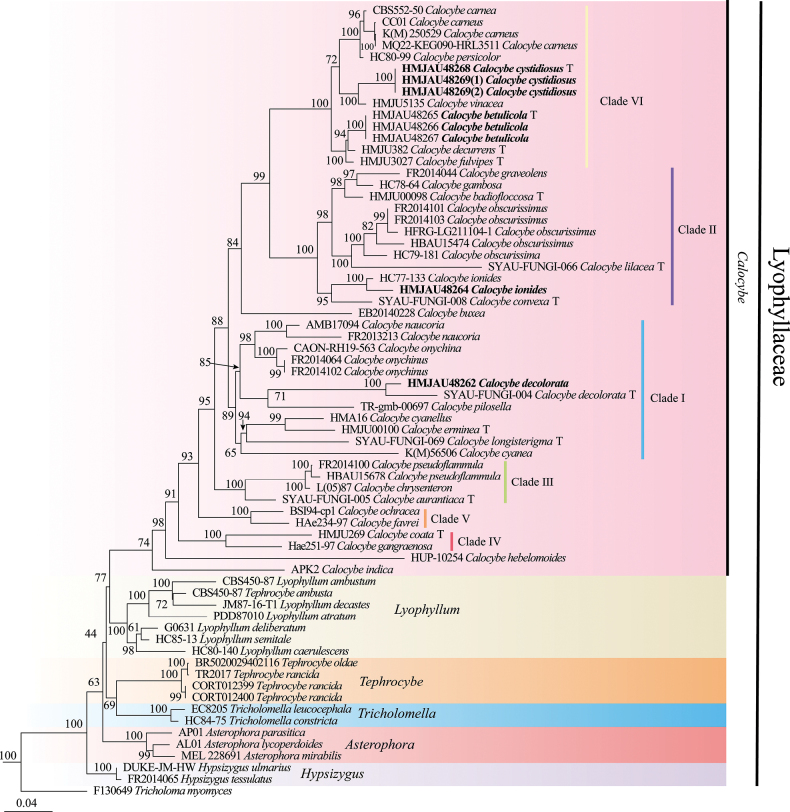
Maximum likelihood phylogenetic tree generated from the ITS, nLSU and *tef1-α* dataset. Bootstrap values ≥ 75% from ML analysis are shown on the branches. Newly sequenced collections are indicated in bold, and the type specimens are denoted by (T).

The concatenated matrix contained 106 sequences (40 for nLSU, 58 for ITS, and eight for *tef1-α*) representing 61 samples were used to build a phylogenetic analysis (the concatenated matrix was deposited at treebase under the acc. no. S31166). Modelfinder selected the best-fit model for the combined dataset, and the best fit model for BI is GTR+F+I+G4. The results of the Bayesian analysis (Fig. 1) and the maximum likelihood analysis (Fig. 2) are generally in agreement.

After trimming, the combined ITS, nLSU, and *tef1-α* dataset represented 46 taxa and 3120 characters. The Bayesian analysis was run for two million generations and resulted in an average standard deviation of split frequencies of 0.009440. The same dataset and alignment were analyzed using the ML method. Six clades were revealed within Lyophyllaceae, representing *Calocybe*, *Tricholomella* Zerova ex Kalamees, *Tephrocybe* Donk, *Asterophora* Ditmar, *Lyophyllum*, and *Hypsizygus* Singer (Figs 1 and 2). Moreover, from our results, the genus *Calocybe* was split into six independent clades, representing five sections and one newly recognized clade. Five sampled specimens formed two independent clades, representing two new species, *C.betulicola* and *C.cystidiosa*.

### ﻿Taxonomy

#### ﻿ Calocybe betulicola

Taxon classificationFungiAgaricalesLyophyllaceae

J.J. Hu, A. Ma, B. Zhang & Y. Li

C15B196E-4A5F-57C4-8B6B-588C30976FD0

Fungal Names: FN 571739

Figs 3 , 4D

##### Etymology.

“*betulicola*” refers to this species that grows on the leaf litter of *Betula* forests.

**Figure 3.**
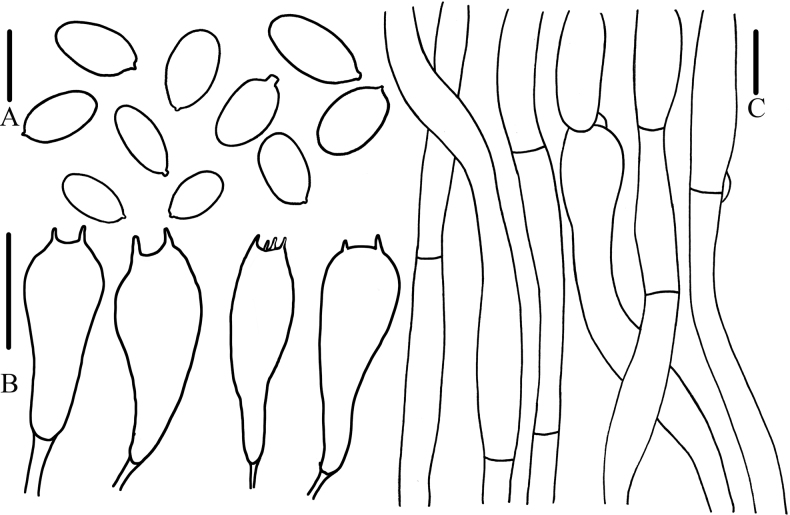
Microcharacteristics of *Calocybebetulicola***A** basidiospores **B** basidia **C** pileipellis. Scale bars: (**A**) 5 μm; (**B, C**) 10 μm.

##### Diagnosis.

This species differs from other species by its grayish-purple pileus, grayish-brown to dark purple stipe, non-cellular pileipellis, and grows on the leaves’ litter of *Betula* forest.

**Figure 4.**
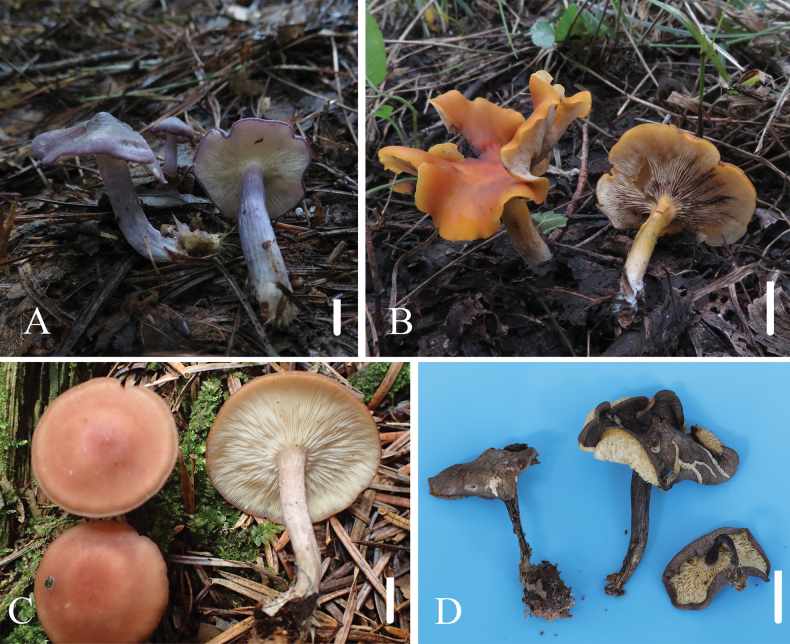
Habitat of *Calocybe* species in this study **A***Calocybeionides***B***Calocybedecolorata***C***Calocybecystidiosa***D***Calocybebetulicola*. Scale bars: 1 cm (**A–E**).

##### Type.

China. Jilin Province, Changchun City, Jilin Agricultural University, 20 September 2021, Jia-Jun Hu and Gui-Ping Zhao, HMJAU48265 (Collection No.: Hu J.J. 1089).

##### Description.

Basidiomata gregarious, small. Pileus convex with an umbo, 2.0–3.5 cm diameter, smooth, violet (18F6) entirely; margin entire, wavy, involute, or reflex occasionally. Lamellae subdecurrent, beige (4B5) to light yellow (30A4), entire, crowded, with 1–3 lamellulae. Stipe cylindrical or tapering downwards, 1.5–3.0 cm long and 0.5–0.8 cm wide, central, with longitudinal stripe, solid, smooth, grayish-brown (18F6) to dark purple (20F7). Context thin, concolor or paler with pileus, odorless.

Basidiospores (2.0)3.0–6.0 × (2.0)3.0–4.0 μm, Q = (1.25)1.33–2.35(2.50), Qm = 1.90, hyaline, oval, smooth, inamyloid, thin-walled. Basidia 10.0–19.0 × 4.0–6.0 μm, clavate, 2- or 4-spored, hyaline, thin-walled. Hymenophoral trama regular and hyphae arranged parallel, not pigmented, hyaline, thin-walled. Pileipellis hyphae 4.0–7.5 μm wide, smooth, hyaline, thin-walled. Stipitipellis hyphae 3.8–9.0 μm wide, hyaline, thin-walled, not pigmented. Clamp connections present.

##### Habitat.

Growing on the leaf litters in birch forests.

##### Additional specimens examined.

China. Jilin Province, Changchun City, Jilin Agricultural University, 18 September 2022, Jia-Jun Hu and Lei Yue, HMJAU48266; Jilin Province, Changchun City, Jilin Agricultural University, 27 September 2023, Lei Yue, HMJAU48267.

##### Comments.

*Calocybebetulicola* is characterized by its grayish-purple pileus, grayish-brown to dark purple stipe, smaller basidiomata, non-cellular pileipellis, and its growth on the leaf litter in birch forests. According to these characteristics, *C.betulicola* is a member of Sect. Carneoviolaceae. Sect. Carneoviolaceae mainly includes four other species, viz. *Calocybedecurrens* J.Z. Xu & Yu Li, *Calocybefulvipes* J.Z. Xu & Yu Li, *Calocybeionides* (Bull.) Donk, and *Calocybecoacta* J.Z. Xu & Yu Li.

This species is macroscopically similar to *C.ionides* due to the purple basidiomata. However, *C.betulicola* differs from *C.ionides* in terms of its unique habitat, subdecurrent lamellae, and wider basidiospores. *Calocybedecurrens* has an intimate affinity in phylogenetic analysis. However, it differed from *C.betulicola* by the gradual fading from pinkish purple to brownish red to grayish brown stipe, carneous pileus, and larger basidiospores ((5.8) 6.0–8.5 (9.3) × (2.1) 2.7–3.8 (4.3) μm) (Xu et al. 2021b). *Calocybefulvipes* differs by its tone brown to dark violet stipe, and the changes it undergoes when injured, bigger Qm, and slightly longer sterigmata (Xu et al. 2021a). *Calocybecoacta* can be distinguished from *C.betulicola* by its cream-gray pileus, the presence of hymenial cystidia, and larger basidiospores (Xu et al. 2021a).

#### ﻿ Calocybe cystidiosa

Taxon classificationFungiAgaricalesLyophyllaceae

A. Ma, J.J. Hu, B. Zhang & Y. Li

181A3CAF-E3D2-5A72-BF59-75C73AEB1D94

Fungal Names: FN 571740

Figs 4C , 5

##### Etymology.

“cystidiosa” refers to the presence of cheilocystidia.

##### Diagnosis.

This species is differentiated from other species by its fresh-pink basidiomata, uncurved margin of the pileus, whitish pink stipe covered with tomentose at the base, lager basidiospores, and the presence of cheilocystidia.

**Figure 5.**
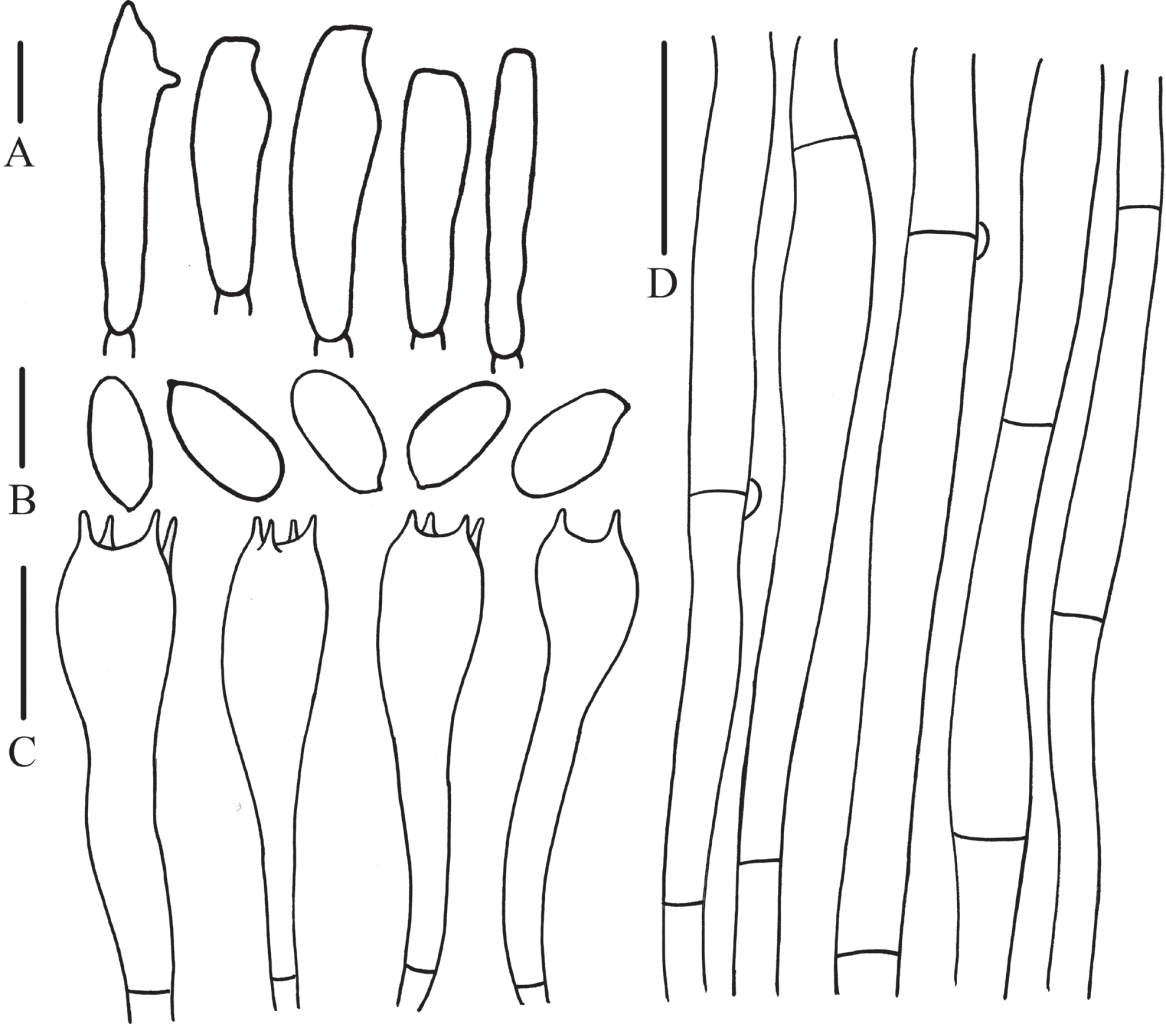
Microcharacteristics of *Calocybecystidiosa***A** cheiocystidia **B** basidiospores **C** basidia **D** pileipellis. Scale bars: 5 μm (**A, B**); 10 μm (**C, D**).

##### Type.

China. Liaoning Province, Fushun City, Xinbin Manchu Autonomous County, Gangshan Provincial Forest Park, Fushun City, August 28, 2018, Ao Ma, HMJAU48268.

##### Description.

Basidiomata solitary to gregarious, small to medium. Pileus 1.8–3.7 cm diameter, convex when young, plane and umbonatus when mature, smooth, dull, flesh-pink (7B4), entire; margin entire, inrolled to incurved. Lamellae white (7A1) to cream (30A2), subdecurrent, adnate, crowded, with a serious lamellulae. Stipe 2.8–4.5 cm long and 0.3–0.6 cm wide, central, paler pink (7B3) to pink (7B44), white (7A1) at apex, solid when younger, then becoming hollow, cylindrical, smooth, fibrous, slightly enlarged towards the base, with white tomentose at base. Context white (7A1), thin, odorless, tastes mild and not distinctive.

Basidiospores (4.0)5.0–6.5(6.9) × (2.0)2.1–2.5 μm, Q = (2.00)2.27–3.00(3.10), Qm = 2.58, hyaline, oval, smooth, inamyloid, thin-walled. Basidia 22.0–28.0 × 5.0–7.0 μm, clavate to cylindrical, 2- or 4-spored, hyaline, thin-walled. Hymenophoral trama regular and hyphae arranged parallel, not pigmented. Cheilocystidia 13.0–20.0 × 3.0–6.0 μm, clavate with an umbo occasionally, or bifurcated, hyaline, thin-walled. Pileipellis hyphae wide 5.0–12.0 μm diameter, smooth, hyaline, thin-walled. Stipitipellis hyphae 3.8–9.0 μm diameter, hyaline, thin-walled. Clamp connections present.

##### Additional specimens examined.

China. Liaoning Province, Fushun City, Xinbin Manchu Autonomous County, Gangshan Provincial Forest Park, Fushun City, 23 June 2018, Ao Ma, HMJAU48269.

##### Habitat.

Grows on the leaf litter in coniferous forests.

##### Comments.

This species is characterized by its growth on the leaf litter in coniferous forests, flesh-pink pileus, fibrous stipe covered with white tomentose at the base, non-cellular pileipellis, larger basidiospores, and the presence of cheilocystidia. These characteristics suggest that *C.cystidiosa* belongs to Sect. Carneoviolaceae according to Singer’s opinion (Singer 1986).

This species is closely related to *C.carnea* due to its pinkish pileus. However, this species can be distinguished from *C.carnea* by its unique habitat, deep color of basidiomata, light yellow lamellae, and larger basidiospores. In the Sect. Carneoviolaceae, *C.vinacea* J.Z. Xu & Yu Li is another species recorded from China with pinkish basidiomata. However, *C.vinacea* differs from this species by the curved margin of pileus, white stipe, smaller basidiospores, and the absence of cystidia (Xu et al. 2021b).

#### ﻿ Calocybe decolorata

Taxon classificationFungiAgaricalesLyophyllaceae

X.D. Yu & Jia J. Li

830DBAED-CD65-5347-8549-C6814AAB6F10

Figs 4B , 6

##### Description.

Basidiomata scattered or gregarious, small to medium. Pileus 1.3–5.0 cm diameter, convex to applanate, involute then becoming reflex, orange-brown (7C8) at center, paler outwards, smooth, hygrophanous; margin petaloid, wavy, orange (6B8). Lamellae subdecurrent, close, white (6A1) at first, black (6E2) at the base to the three-quarter towards the margin when mature, with 1–5 lamellulae, edge denticulate. Stipe 2.3–4.2 cm long and 0.3–0.9 cm wide, central, cylindrical, or enlarged at apex, light orange-brown (6A6), with green tone at center, covered with white tomentose at base, hollow when mature. Context fleshy, thin, odorless.

**Figure 6.**
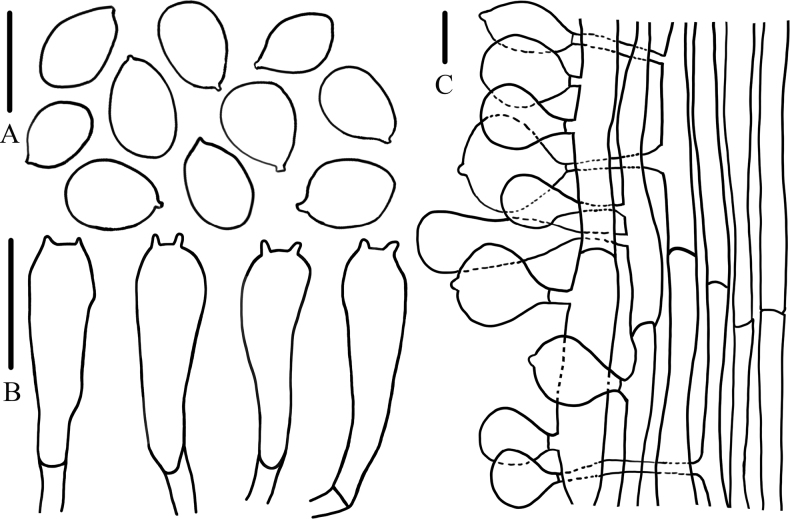
Microcharacteristics of *Calocybedecolorata***A** basidiospores **B** basidia **C** pileipellis. Scale bars: 5 μm (**A**); 10 μm (**B, C**).

Basidiospores (2.0)2.9–5.0 × (1.5)2.0–3.2 μm, Q = (1.15)1.17–1.50(1.60), Qm = 1.34, subglobose, hyaline, inamyloid, smooth, thin-walled. Basidia 11.1–21.5 × 3.7–6.0 μm, clavate, 2-spored, occasionally 4-spored, hyaline, thin-walled. Hymenophoral trama regular and hyphae arranged parallel, not pigmented, 2–3 μm wide. Pileipellis an epicutis composed of dense, radially parallel, hyphae 2.5–11.3 μm in width, smooth, hyaline, terminal cells a bulbous shape. Stipitipellis hyphae smooth, pigmented, 2.5–8.8 μm diameter.

##### Specimen examined.

China. Jilin Province, Changchun City, Jilin Agricultural University, 21 Aug 2019, Jia-Jun Hu and Gui-Ping Zhao, HMJAU48262 (Collection no.: Hu J.J. 591).

##### Habitat.

Grows on the leaves’ litter in broad-leaved forests.

##### Comments.

This species was originally described from Liaoning Province, China by Li et al. (2017) and is mainly characterized by a brighter orange or yellow color pileus, light orange-brown stipe, and smaller basidiospores. The species was classified as a species of Sect. Carneoviolaceae based on its main morphological characteristics.

However, there are some differences between our specimen and the type specimen. The specimens observed in this study have bulbous-like terminal hyphae in the pileipellis, which were not described in the type species.

#### ﻿ Calocybe ionides

Taxon classificationFungiAgaricalesLyophyllaceae

(Bull.) Donk

771F7766-2249-5509-BCA1-63148578BEB1

Figs 4A , 7

##### Description.

Basidiomata gregarious, small. Pileus 1.3–2.8 cm diameter, convex to oblate semispherical, with an umbo at center, hygrophanous, smooth, entire, involute, violet (16E8) to purple-black (17E8), occasionally deeper at center. Lamellae white (16A1), crowded, adnate, with 1–3 lamellulae. Stipe 1.5–3.0 cm long and 0.1–1.2 cm wide, center, paler violet (16E8), cylindrical, hollow, smooth, fibrous, covered with white tomentose at base. Context thin, white, fleshy, odorless.

**Figure 7.**
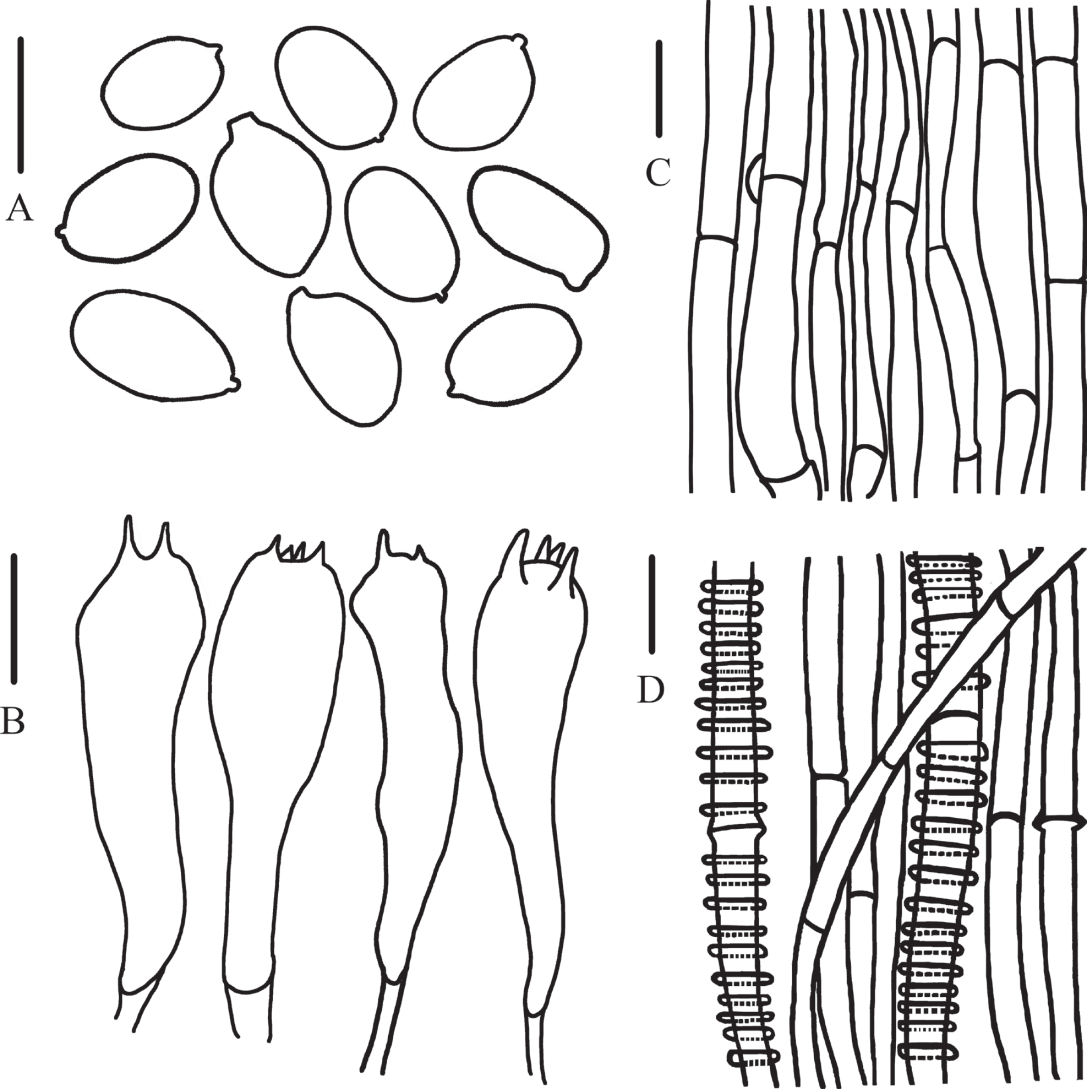
Microcharacteristics of *Calocybeionides***A** basidiospores **B** basidia **C** pileipellis **D** stipitipellis. Scale bars: 5 μm (**A**); 10 μm (**B–D**).

Basidiospores (3.0)4.0–6.0 × (2.0)2.2–3.0 μm, Q = (1.50)1.67–2.40(2.50), Qm = 2.11, oblong, smooth, hyaline, inamyloid. Basidia 12.0–19.0 × 3.0–6.0 μm, clavate, 2- or 4- spored, hyaline, thin-walled. Pileipellis hyphae 3.0–6.0 μm wide, smooth, hyaline. Stipitipellis hyphae smooth, 3.0–7.5 μm wide, annulated, with a litter thick-walled.

##### Specimen examined.

China. Jilin Province, Changchun City, Jingyuetan National Forest Park, 27 Aug 2019, Jia-Jun Hu and Gui-Ping Zhao, HMJAU48264; Liaoning Province, Fushun City, Xinbin Manchu Autonomous County, Gangshan Provincial Forest Park, 13 September 2018, Ao Ma, HMJAU 49165; Heilongjiang Province, Da Hinggan Ling Prefecture, Shuanghe National Nature Reserve, 18 July 2019, Di-Zhe Guo, HMJAU 48270.

##### Habitat.

Grows on the leaf litter in coniferous or broad-leaved forests.

##### Comments.

The main characteristics of this species are small basidiomata, a purple-blue color of the pileus, white lamellae, and a stipe that is either of the same color or lighter than the pileus. According to its main morphological characteristics, this species can be assigned to Sect. Carneoviolaceae.

### ﻿Key to the reported species of *Calocybe* from northeast China

**Table:** 

1	Pileus with orange to gray-brown tones, usually grows on coniferous forest, or mix	**3**
–	Pileus without orange-yellow to gray-brown tones, usually grows on broad-leaved forest	**2**
2	Pileus with pink to red tones	**7**
–	Pileus without pink to red tones	**11**
3	Lamellae blue when bruised, cystidia present	***C.decolorata***
–	Lamellae color unchanged when bruised, cystidia usually absent	**4**
4	Lamellae yellow, covered with dense white fibrils at base	***C.aurantiaca***
–	Lamellae not yellow, not covered with dense white fibrils at base	**5**
5	Pileipellis cellular, basidiospores subglobose	***C.erminea***
–	Pileipellis noncellular, basidiospores not subglobose	**6**
6	Pileus felty, sterigmata shorter than 5 µm	***C.coacta***
–	Pileus not felty, sterigmata longer than 5 µm	***C.longisterigma***
7	Pileus dull-red, color of stipe not similar with pileus	***C.vinacea***
–	Pileus not dull-red, color of stipe similar or paler than pileus	**8**
8	Habitat is white birch forest, basidiomata grows on leaf litter of *Betula*	***C.betulicola***
–	Habitat not white birch forest, basidiomata does not grow on leaf litter of *Betula*	**9**
9	Lamellae grayish-orange when bruised, stipe usually smooth	***C.fulvipes***
–	Lamellae unchanged, greyish-orange when bruised, stipe not smooth	**10**
10	Stipe turn purple when mature, cystidia not present	***C.decurrens***
–	Stipe does not turn purple when mature, cystidia present	***C.cystidiosa***
11	Pileus with purple tones, pileipellis a trichoderm	***C.ionides***
–	Pileus without purple tones, pileipellis not trichoderm	**12**
12	Stipe with white pubescence at base, basidiospores biger than 5 µm	***C.badiofloccosa***
–	Stipe without white pubescence at base, basidiospores shorter than 5 µm	***C.convexa***

## ﻿Discussion

The genus *Calocybe* exhibits a wide distribution in China, but the full extent of its species diversity remains uncertain. This study provides a detailed description of two new species, namely *C.betulicola* and *C.cystidiosa*, as well as one previously unrecorded species, *C.decolorata*, found in Jilin Province. Additionally, a common species, *C.ionides*, was also identified in northeastern China. Moreover, the phylogenetic analysis confirmed all of the species that were previously reported.

The phylogenetic analysis, based on the combined ITS, nLSU, and *tef1-α* dataset, revealed that Lyophyllaceae forms a monophyletic clade. Moreover, the Lyophyllaceae clade was divided into six subclades, representing six independent genera, viz. *Calocybe*, *Lyophyllum*, and *Tricholomella*, etc. In addition, the genus *Calocybe* forms a monophyletic clade with “*Rugosomyces*”, consisting of Bellanger et al. (2015), Li et al. (2017), and Xu et al. (2021a). Thus, the demarcation between the genus *Calocybe* and other genera within the Lyophyllaceae family is more distinct.

However, our phylogenetic analysis reveals certain discrepancies when compared to the findings of Li et al. (2017) and Xu et al. (2021a). In the present study, we identified six distinct sectional clades within the genus *Calocybe*, supported by robust evidence. These clades have been designated as clade I to clade VI. Notably, a new sectional clade, referred to as clade VI, has been identified for the first time in this study. This clade (clade VI) is featured by the presence of a pinkish to reddish pileus and primarily consists of two newly discovered species, namely *C.carnea*, and *C.persicolor*, etc.

In addition, Clade I consists of *Calocybeonychina* (Fr.) Donk, *Calocybenaucoria* (Murrill) Singer, and *Calocybeerminea* J.Z. Xu & Yu Li, etc., distinguished by a pileus that ranges in color from white to yellow. The Clade II comprises primarily of *Calocybeobscurissima* (A. Pearson) M.M. Moser, *Calocybelilacea* X.D. Yu, Ye Zhou & W.Q. Qin, *Calocybegraveolens* (Pers.) Singer, etc., characterized by pileus color ranging from white, yellow to violet shades. The Clade III consistent with *Calocybechrysenteron* (Bull.) Singer, *C.aurantiaca*, and *Calocybepseudoflammula* (J.E. Lange) M. Lange ex Singer, and is characterized by a yellow pileus. The main distinguishing characteristics of Clade IV, which includes *C.gangraenosa* and *C.coacta*, are the white-colored to grayish-yellow pileus. The Clade V is distinguished by the presence of a gilded pileus and includes two species, *Calocybeochracea* (R. Haller Aar.) Bon and *Calocybefavrei* (R. Haller Aar. & R. Haller Suhr) Bon.

Based on the findings of the present study, we increased the species diversity of the genus *Calocybe* in China. The taxonomic system of this genus remains a subject of debate due to insufficient species sampling and the inadequate genetic variation in the DNA loci. Therefore, additional evidence is needed to contribute to a more comprehensive understanding of the genus. Furthermore, despite the recent identification of new species of *Calocybe* from northeast China, the true extent of its species diversity remains uncertain and calls for a comprehensive systematic analysis.

## Supplementary Material

XML Treatment for Calocybe betulicola

XML Treatment for Calocybe cystidiosa

XML Treatment for Calocybe decolorata

XML Treatment for Calocybe ionides

## ﻿Additional information

### Author ORCIDs

Ao Ma https://orcid.org/0000-0001-8635-9767

Jia-Jun Hu https://orcid.org/0000-0002-7562-7612

Yong-Lan Tuo https://orcid.org/0000-0001-6019-1038

Xue-Fei Li https://orcid.org/0009-0005-2556-6494

Dan Dai https://orcid.org/0000-0002-9642-2480

Bo Zhang https://orcid.org/0000-0001-9508-8188

Yu Li https://orcid.org/0000-0003-4719-7210

### Data availability

All of the data that support the findings of this study are available in the main text.
